# Notes of a Few Cases of Hernia

**Published:** 1853-11

**Authors:** T. J. Orr

**Affiliations:** Cincinnati


*Notes of a few Cases of Hernia.* Treated by T. J. Orr, Cincinnati.

A. D., aged forty-seven years, very corpulent, weighing over three hundred pounds, had been ruptured at the umbilicus for about three months ; the rupture was through the lower margin of the ring, extending down the linea alba about ten lines, forming with the ring a triangular opening. The tumor when fully protruded, was about eighteen lines in diameter, very tense, but could be readily reduced by pressure with the fingers.

May 14th.—Returned the bowel and applied a pyrmidal or nipple-shapped pad, corresponding in its lateral diameters to the irregular form of the herniary opening, and of such a size as to pass in firmly against its edges, being prevented by a round shoulder on the body of the pad from sinking in deeper than the thickness of the wall of the abdomen.

July 17th.—Removed the instruments, and found the skin very red and tender to the touch, the discoloration about the navel being about the size of a dollar, and supposing the inflamation had terminated in the effusion of lymph, we applied the "cup pad," formed like the preceeding, but excavated in its centre to such a width as to reach a line or two beyond the margin of the opening, and to such a depth as to contain only, and be completely filled by, the skin and the other coverings of the hernia—wider at its mouth than at the bottom, so as not to strangulate the parts included.

July 27th.—On removing the instrument, the navel presented the appearance of a large rivet head—an exact cast of the cup in the pad, the redness had entirely disappeared from the part contained in the excavation, but a brignt areola remained outside of the boundary marked by the cup. The part was washed with cold alum water, and the instrument re-applied.

July 10th.—Lifted the pad off and examined the protuberance which was of the same color and temperature of the healthy skin, but felt like a solid cartilaginous knob, perfectly aglutinated together down to the ring. There was no impulse imparted to the finger when the patient coughed.

The cupped pad was now laid aside, and one slightly convex applied, which, in ten days, flattened down the projection and completed the cure.

Case 2nd.—Mr. T. J., aged forty-five, a merchant of delicate habit and very relaxed fibre, had hernia of both sides for ten years; general health much impaired, symptoms, those of inveterate dyspepsia. The tumor of the right side from the external ring to the testicle, occupied the entire scrotum, putting it considerably on the stretch, internal ring dragged within half an inch of the external, both greatly enlarged and relaxed. The sac was thickened by frequent occurrence of inflamation in the tumor. On the patient lying down the bowel would pass immediately back into the cavity of the abdomen, and again protrude in coughing, turning in bed, or by almost the slightest movement of the body, keeping up in this way an almost incessant irritation of the digestive organs.

The tumor of the left side, about the size of an egg, was the the common inguinal form, had not passed through the external ring.

May 5th.—Commenced the treatment by reducing the tumors and applying a double instrument with the nipple pads, the elevation on the face of the pad for the right side, was cut to the depth of three or four lines, wide enough, and of such a figure as to cover accurately the hernial apperture, for the left side, the projection was of about the same depth, but longer as the rings were the usual distance apart. These pads so formed, that their lower margins correspond exactly with Pupart's ligament, the projections from their face resting over the centre of the inguinal passage.

May 20th—Removed the truss and found under each pad a red spot of the same shape and size of the projection on the pad; directed a cold wash for the part, and the instrument to be re-applied.

June 10th.—Examined the patient, the part of the pad was entirely burried in the integuments, forming a deep indentation which was very red and tender. The nipple pads were now removed, and the cupped ones took their place, their cavities so formed as to exactly cover the depression caused by the pressure of the former.

July 1st.—Found that the inflamed depression in the wall of the abdomen had mounted up into the cups in the pads and so completely packed in as to impart to the touch the sense of much greater density than the surrounding parts. The skin covering the eminence had entirely lost its redness, was in fact paler than elsewhere, having an appearance of the effusion of serum under the cuticle. Ordered the use of an astringent lotion, and the pads returned into precisely the same track.

July 15th.—Found no appreciable change, the instrument was worn from this time until the fifteenth of August, when the case appeared to be radically cured, but for fear it might return, an instrument of about half the strength, with flat pads was substituted and worn for thirty days, when the patient was able to dispense with it altogether.

Case 3d.—Master George, a lad of eight years old, had scrotal hernia of the right side, caused by a paroxism of whooping-cough, the bowel was projected through the inguinal passage with great force, so as to reach a point quite below the posterior margin of the scrotum, where it lodged between the skin and perineal facia near the anus.

This case may be said to be the same as if it had been congenital, for up to the time of the protrusion of the gut, there had been but one testicle (*i. e.* the left one), the bowel could be stripped back with the fingers through the abdominal rings, leaving behind the sac which was evidently the tunica vaginalis, and the other coverings of the testicle perfectly empty, or at least containing nothing but the mere rudiments of that gland.

The treatment in this case, after the bowel had been reduced, consisted, first in the use of the pyramidal pad for thirty days, followed by the cupped one, which was worn about the same length of time, when the instrument was taken off, since which (being over five months) there has been no appearance of the disease up to the present time.

[We have examined a number of the cases of Hernia that have been treated by Dr. Orr's peculiar method; and we are perfectly satisfied that his success entitles him to the confidence of the profession. There is no department of Surgery in which there is so much quackery as in the management of Hernia, and in this city it has been allowed to go too much into the hands of unprincipled charlatans, who have no knowledge of the anatomy of the parts involved, or the proper mechanical means to be used for their relief. We are therefore pleased to see a man of Dr. O.'s integrity and qualifications, making Hernia a speciality.]

